# 2020 COVID-19 lockdown and the impacts on air quality with emphasis on urban, suburban and rural zones

**DOI:** 10.1038/s41598-021-99491-7

**Published:** 2021-10-29

**Authors:** Klara Slezakova, Maria Carmo Pereira

**Affiliations:** LEPABE, Departamento de Engenharia Química, Faculdade de Engenharia, Universidade do Porto, Rua Dr. Roberto Frias, 4200-465 Porto, Portugal

**Keywords:** Environmental sciences, Environmental impact

## Abstract

Air quality improvements pollution changes due to COVID-19 restrictions have been reported for many urban developments and large metropolitan areas, but the respective impacts at rural and remote zones are less frequently analysed. This study evaluated air pollution changes across all Portugal (68 stations) considering all urban, suburban and rural zones. PM_10_, PM_2.5_, NO_2_, SO_2_, ozone was analysed in pre-, during, and post-lockdown period (January–May 2020) and for a comparison also in 2019. NO_2_ was the most reduced pollutant in 2020, which coincided with decreased traffic. Significant drop (15–71%) of traffic related NO_2_ was observed specifically during lockdown period, being 55% for the largest and most populated region in country. PM was affected to a lesser degree (with substantial differences found for largely populated areas (Lisbon region ~ 30%; North region, up to 49%); during lockdown traffic-related PM dropped 10–70%. PM_10_ daily limit was exceeded 50% less in 2020, with 80% of exceedances before lockdown period. SO_2_ decreased by 35%, due to suspended industrial productions, whereas ozone concentrations slightly (though not significantly) increased (83 vs. 80 µg m^–3^).

## Introduction

In January 2020 the World Health Organization (WHO) declared a global health emergency because of the novel coronavirus disease (COVID-19) that has been uncontrollably spreading all over the world^1^. Since then, the pandemic has affected our whole society; WHO has registered 123 million cases worldwide with a total of 2.7 million mortalities^2^. To limit the spread of pandemic, governments in countries around the world have imposed various restrictions, which led to reduction of people movement, decrease in transport (road and aviation), and even suspended industrial activities^3^. While COVID-19 has caused many adverse changes to our society and economies^4,5^ and even to environment (in a form of newly created medical waste^6^), some studies have emphasized a possible improvement of the state of the environment^7^. People confinement, restricted public transport and ceased airlines international flights have also resulted in changes in air pollutant emissions, with information for megacities (Rio de Janeiro and Sao Paolo^8,9^; and highly populated urban zones and cities^10–17^. On more global scale, during the COVID-19 pandemic, PM_2.5_ dropped approximately 12% across the most polluted cities worldwide, with the greatest reduction in capitals of America, Asia and Africa^18^. While the current studies even show that there might be causality between air pollution and COVID-19 infection spread^19,20^, the main focus of the COVID air pollution studies is typically on large and densely populated urban areas^21–23^ during relatively a short (or part of) period of lockdowns. The changes in air pollution trends during the lockdown in rural or remote places have been reported to much lesser degree. Ceased air and road transport and restricted human movements during the lockdowns allow for a unique situation for prediction of modelling and visualization of potential air pollution mitigation scenarios. However, the complete perspective requires first information on air pollution changes not only in urban but also in rural and remote areas. This work thus evaluates the air pollution evolution (PM_10_, PM_2.5_, NO_2_, SO_2_, ozone) in pre-, during and post-lockdown period (January 1–May 2020) in whole Portuguese territory (continent and islands; 7 regions) considering all rural, suburban, urban zones. To provide wider context, air pollution data are assessed and also compared to the same period of the previous year.

## Materials and methods

### Air quality network

The assessment of air quality in Portugal is conducted by the Portuguese Environment Agency (APA). The air quality measurement stations are managed by the Regional Development and Coordination Commissions (CCDR) of the region in which they operate. The classification of seven regions of Portuguese territory (five for the continent—North, Centre, Lisbon and Tejo Valley (Lisbon TV), Alentejo, Algarve; two for islands—Madeira, and Azores) was also adopted for this work (Supplementary Table 1S of the Supplementary Information). Each monitoring station is characterized based on its type of agglomeration zone (rural, suburban or urban). The predominant influence of anthropogenic emissions further determines sub-type of each site as traffic, industrial or background. The air pollution data are measured continuously at all monitoring stations from which they are transmitted, in almost real time, to regional “centres”. From these they are communicated to the central information system of QualAr database^24^, based at APA^25^. The data are then made available to the public through QualAr portal^26^. Table 1 and Supplementary Fig. 1S of the Supplementary Information summarized the characteristics of monitoring stations per each district. All 68 monitoring stations were considered in this work.

**Table 1:** Air pollution monitoring network in Portugal: summary of zone- and emission influence-specific monitoring sites in each region.

Region	Stations n (%)	Type n (%)								
		Rural			Suburban			Urban		
		Background	Traffic	Industrial	Background	Traffic	Industrial	Background	Traffic	Industrial
North	22 (32)^a^	3 (14) ^b^	–	–	6 (27)	–	1 (5)	5 (23)	6 (27)	1 (5)
Centre	9 (13)	4 (44)	–	–	2 (22)	–	–	1(11)	2 (22)	
Lisbon TV	24 (35)	3 (13)	–	–	–	–	1 (4)	13 (54)	5 (21)	2 (8)
Alentejo	5 (7)	2(40)	–	1 (20)	1 (20)	–	–	–	–	1 (20)
Algarve	4 (6)	1(25)	–	–	–	–	–	2 (50)	1 (25)	–
Madeira	3 (4)	1 (33)	–	–	–	–	–	1 (33)	1 (33)	–
Azores	1 (2)	1	–	–	–	–	–	–	–	–
Total	68	15	–	1	9	–	2	22	15	4

^a^% considering the whole territory.

^b^Indicated % is estimated considering of abundance of a station/type in each district.

Depending on the density and distribution of buildings, stations are classified as the folloing: rural—all other areas; suburban—largely built-up; urban area urban—continuously built-up urban area; background stations—pollution levels are representative of the average exposure of the general population or vegetation; traffic—situated in a close proximity to a single major road; industrial stations—situated in close proximity to an industrial area or an industrial source^27^.

### Air pollution data

The state of emergency due to the spread of COVID-19 pandemic was enforced from 19 March to 2 May 2020, with a strict consequent phase until 1 June 2020^28^. Up to this date, various limitations are still applied in Portugal (among other restrictions for public gatherings, obligatory use of masks in enclosed public spaces such as transport, shops or public offices, restricted working hours for bars and restaurants, etc.). Air pollution data were thus retrieved from the public QualAr database for all five months of 2020, specifically between January 1 and May 31. The data from the same period of the previous year 2019 were also considered for the comparison.

All air pollutants available online by QualAr were considered, namely, PM_10_, PM_2.5_, O_3_, NO_2_, and SO_2_. For particles, daily (24 h) average concentrations are published whereas it was maximum hourly average for O_3_, NO_2_, and SO_2_. Air quality standards in Portugal^29,30^ are based on the existent European legislation govern by Directive 2008/50/EU^31^; for a reader convenience the respective standards and the limit values are summarized in Supplementary Table 2S).

### Traffic data

There are 15 principal motorways in continental Portugal. As there is no public database that would provide summarized information on number of vehicles in Portugal, the existing data regarding traffic counts were retrieved from available annual reports of the company^32,33^ that ensures the majority operations of motorways system in the country. Additional information was then retrieved from public reports and research projects, published on web portals of city halls of Metropolitan Areas of Lisbon and of Oporto^34–36^ (Supplementary Table 3S). Data on activity across various sectors was obtained from Google LLC^37^.

### Statistical analysis

All statistical tests for this study were performed by SPSS (IBM SPSS Statistics 26) and Microsoft Excel 2013 (Microsoft Corporation). Medians and means were compared through the non-parametric Mann–Whitney U test as the obtained data did not display normal distributions (confirmed by Shapiro–Wilk’s test). Statistical significance was set as *p* < 0.05.

## Results and discussion

### Air pollution monitoring network

As shown in Table 1 and Supplementary Fig. 1S, most of the monitoring sites were situated in the districts of North and Lisbon TV. These two regions accounted for 67% of all monitoring stations while they compose approximately 35% of the Portuguese territory area (Supplementary Table 1S). Centre region accounted for 13% of the monitoring sites, whereas in Alentejo (30% of the total area) and Algarve district (5%) there are 7 and 6% of the monitoring stations, respectively. Portuguese islands represent a much smaller area (~ 3.5%) and hence a limited amount of monitoring stations (6%).

Evaluating the zone impact of each station (i.e. rural vs. suburban and urban) it is noteworthy that monitoring stations in urban zones are predominantly situated in North and Lisbon TV regions (47%). This is understandable considering that these two regions contain the two largest Metropolitan Areas in the country (of Oporto and Lisbon, respectively), with approximately 45% of the total Portuguese population (17 and 28% for area, respectively, in 2019)^38^. In fact, monitoring sites of urban zones account for 55 and 83% in each of these regions, respectively. In addition, the North was the region with the highest number of monitoring sites in suburban zones (10%), whereas the monitoring stations for rural areas are relatively uniformly distributed among all regions (4–6% in North, Centre, Lisbon TV and Alentejo, 1–3% in the remaining regions).

Evaluating the specific emission sources, 40% of all monitoring sites with traffic influence were situated in Northern monitoring network where it consists 28% of all the district stations. In addition, 65% of all background sites were situated in the most populated regions of Lisbon TV (67% the district stations) and in North (63%). On the national perspective, monitoring stations under influence of industrial emissions were the least existent (10%), being uniformly distributed between North, Lisbon TV and Alentejo region.

### Traffic data

Evaluation of daily average traffic (on motorways) in 2018–2020^32,33^ is shown in Supplementary Fig. 2S. It is clear that quarterly (Q1–Q4) evolution trends of traffic were the rather same between 2018 and 2019, with the highest peak always observed during the summer months, i.e. 3rd quarter (Q3, 25,530 vs. 25,916 vehicles day^–1^, respectively). Similarly, this trend also occurred in 2020 (Q3 peaking with 22,060 vehicles day^–1^). Furthermore, when compared to previous year, in 2019 traffic showed a consistent growth of 3.7%, for each quarter as the following, 5.6% for Q1, 6.3% for Q2, 1.5% for Q3 and 2.1% for Q4. It noteworthy, that heavy vehicle traffic showed a higher growth rate (4.5%) than light vehicles (3.6%). In agreement, additional data showed that road transport supply (for transport of passengers) increased to 29.4 billion seats-km, with 83.1% of its total being made available on regular transport^35^. The number of national transport services increased by 9.2% to 20.5 million, while 543.1 million passengers were carried, representing an increase of 5.5% compared to the previous year of 2018^35^.

On the contrary, the overall amount of traffic decreased during all Q1–Q4 of 2020. During the first quarter of 2020 the average daily traffic was 12% lower in a comparison with 2019 and 7% lower when compared with 2018 (Supplementary Fig. 2S). Mobility data (Supplementary Fig. 3S) that is based on historical geo-localization of mobile phones with activated GPS showed that people spent up to 30% more time at home during March–May 2020^37^. The mobility to work and the use of public transportation significantly decreased (up to 66 and 78%)^37^. In addition, due to the restrictions associated with COVID-19, retail and recreational mobility were strongly reduced (up to 76%) as well as visits to grocery and pharmacy shops (up to − 46%)^37^. Specifically, for the Q2 of 2020 the traffic reduction was 46 and 49% when compared with the previous years and it represented 32% decrease (when compared with Q1 of 2020; Supplementary Fig. 2S). Furthermore, the available data show that light vehicles were more impacted by the traffic reductions than heavy vehicles, with the respective decrease of 12 and 2% respectively^33^; in 2020 heavy vehicles accounted for 7.2% of the traffic (vs. 5.7% in 2019, Supplementary Fig. 4S).

### Air pollution data, 2019 vs. 2020

#### Particulate matter

**Figure 1:**
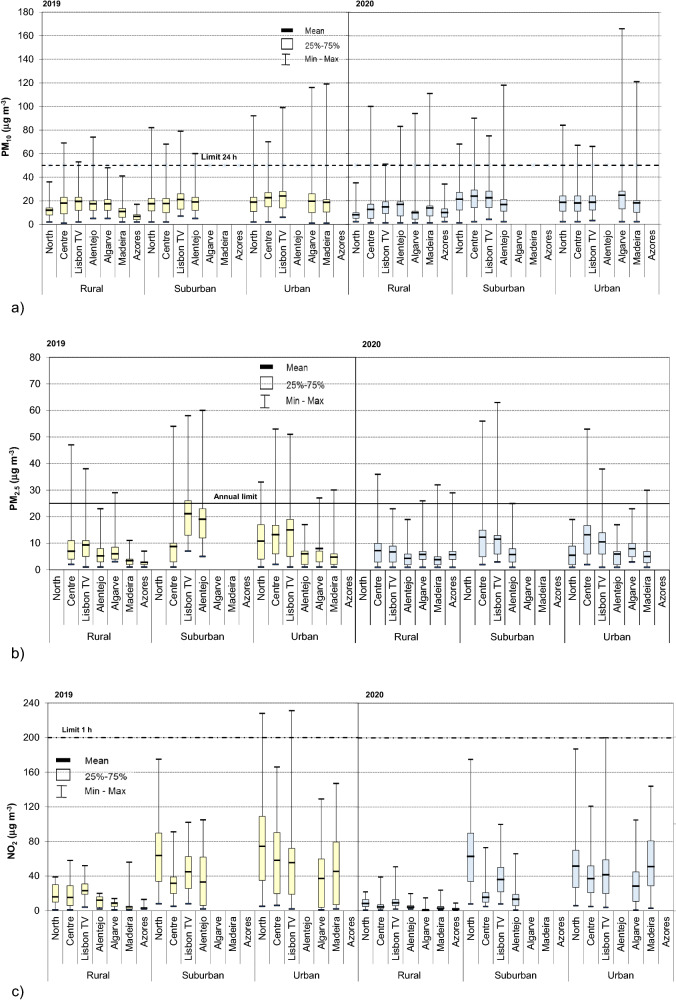

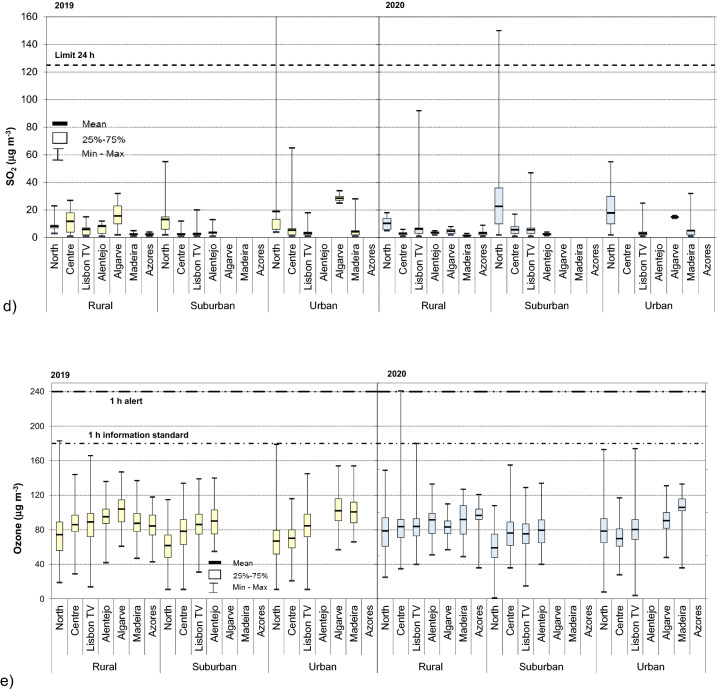
Concentrations of particulate and gaseous air pollutants (closed square, median; open square, 25–75%, and range) in (January–May) 2019 and 2020 in Portugal, (**a**) PM_10_; (**b**) PM_2.5_; (**c**) NO_2_; (**d**) SO_2_; and (**e**) O_3_. Notes: Horizontal dashed lines represent the 1 h and 24 h standards as defined in Directive 2008/50/EU^31^. For PM_2.5_ horizontal line represents annual limit. Distributions of each pollutant were significantly different (*p* < 0.05) across the three zones and emissions sources. The concentrations of PM are expressed as 24 h averages whereas the gaseous pollutants are expressed as 1 h maxima. For better visualization vertical axis y uses different scale.

The levels of air pollutants, namely PM_10_, PM_2.5_, NO_2_, SO_2_ and O_3_ in seven Portuguese regions are summarized Fig. 1, which show the statistics across all monitoring stations for the three types of zoning (rural–urban). Specifically, in 2019 daily levels of PM_10_ measured at 68 monitoring stations demonstrated large variations of the obtained data (Fig. 1a), with detailed descriptive statistics summarized in Supplementary Table 4S. Average daily PM_10_ means were between 12 and 20 µg m^–3^ (absolute range 1–74 µg m^–3^) in rural zone of the five regions of Continental Portugal, 18–21 µg m^–3^ (2–82 µg m^–3^) in suburban zones and 20–24 µg m^–3^ (1–116 µg m^–3^) in urban ones. Regions of Portuguese islands showed lower concentrations, especially at rural zones with the corresponding means of 11 µg m^–3^ (Madeira, range 2–71 µg m^–3^) and 7 µg m^–3^ at Azores (2–17 µg m^–3^). These results demonstrated that for all 7 regions daily PM_10_ concentrations were the lowest at rural zones being significantly (*p* < 0.05) different (approximately 15% in Algarve—60% for North) that the respective means at urban zones (or suburban for Alentejo). Regarding the EU limits daily (50 µg m^–3^), for urban zones the exceedances were observed in 30% of the rural stations, two in Centre, one in Lisbon TV, one in Madeira, one in Alentejo (the respective monitoring station is with industrial influence due to the industrial power plan). It is though necessary to point out that EU legislation stipulates a tolerance of exceedance 35 per year and in that regard all the monitoring stations fulfilled the conditions as the registered exceedances occurred 1–7 times per the same station (Supplementary Table 5S). Furthermore, it is necessary to enhance that the raw data were considered in this work and the possibility of subtracting contributions to the measured concentrations from natural sources and winter road sanding/salting has not been considered. In all the other regions (North, Algarve and Azores islands), no concentrations higher than daily limit were registered in rural zones. On the contrary, suburban and urban zones of Portugal exhibited exceeded the daily limits in all regions/zones. These were especially high for North (total of 57) and in Lisbon TV (64), which were the regions with the higher number of stations. In Centre, the limits were approximately 3 times less (21 times), whereas in Alentejo and Algarve the exceedance were even less frequent (6 and 12 times, respectively); in all stations the margin of tolerance (35 exceedance) fulfilled in all monitoring sites.

In 2020 the levels of PM_10_ (Fig. 1a, Supplementary Table 5S) were slightly lower in a comparison with the previous year. Daily means of PM_10_ for Continental Portugal were observed as the following, 8 µg m^–3^ (North) to 17 µg m^–3^ (Algarve) in rural zones, 17 µg m^–3^ (Alentejo)—24 µg m^–3^ (Centre) in suburban one, and 17 µg m^–3^ (North) and 25 µg m^–3^ (Algarve) for urban zones. These results showed that in rural zones, PM_10_ concentrations in 2020 were significantly lower than in the previous year (p < 0.05; overall mean of 12 µg m^–3^ vs. 17 µg m^–3^ in 2020), with the respective percentage being between 30% (Lisbon TV) and 80% in Alentejo. In urban zones, the respective PM were lower in 2020 only in Centre and Lisbon TV regions (~ 30%), whereas PM no differences were observed in North and Alentejo region. Within the urban areas, ambient air pollution is typically dominated by motor vehicles traffic, but due to the variables such as number of junctions, distance to roadways, traffic flows, surrounding road length, and others the respective pollution levels may vary greatly^39–41^. It is assumed that the lesser traffic in 2020 (Supplementary Fig. 2S) might be the cause for the lower PM levels in some of the urban zones.

**Figure 2:**
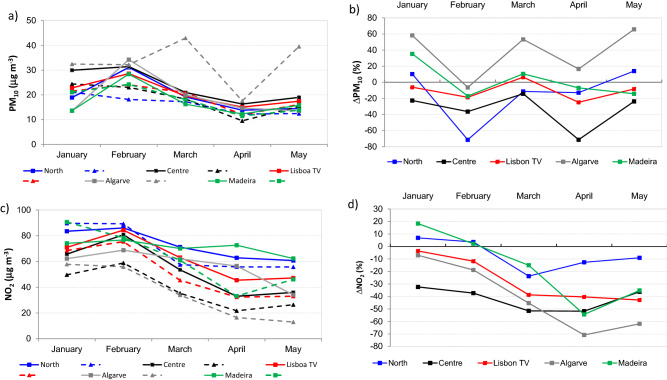
Assessment of traffic related pollution of PM_10_ and NO_2_, (**a**), (**c**) monthly profiles (January–May) in 2019 and 2020; (**b**) and (**d**) representations of pollutant concentration changes in 2020 vs*.* 2019. Dashed lines represent 2020 values whereas continuous lines represent levels in 2019. Monthly means were estimated across 22 urban/traffic monitoring stations.

The analysis of PM_10_ levels across the urban zones with traffic emissions specifically (i.e. 22 monitoring urban-traffic sites) showed that in terms of monthly evolution (Fig. 2a) PM_10_ started to decrease in February 2020 (mainly North and Centre), with the minimal levels (in all regions) observed in April (when the state of emergency was still implemented) and then increased in May (state of emergency ended).The examples of temporal variations of the pollutants are shown in Supplementary Fig. 5S–7S. During the lockdown period, PM_10_ showed a decline (for all the regions), having the highest reductions in the Centre region (61%). The reduced vehicular movement, limited industrial and construction activities could be responsible for the decline in PM_10_ emissions^42^.

When comparing concentrations at urban traffic sites between the both years (Fig. 2b), the largest drops of PM_10_ were observed, as expected in month of April (10–70% in Madeira and Algarve) but also February (up to 70% in North). These data may are in agreement with the road transport trend that shows overall lower traffic in 2020 (during the first and second trimester; Supplementary Fig. 2S–3S). Furthermore, it needs to emphasized that though in urban areas, PM_10_ is strongly affected by local emissions (e.g., traffic including resuspension, building works, industry, etc.), meteorology is a significant parameter for pollutant levels and dispersion. Therefore, analysis of meteorological parameters (temperature, precipitation, wind directions and intensity, pressure distribution) would be precious in order to assess the role of the meteorology on the observed concentration changes and the impact of the emission reductions. In general, the obtained results are in agreement with study by Gama et al.^43^ who reported PM_10_ levels at selected sites across Portugal for that respective period. Authors reported overall drop of 18% (a mean reduction of about 16% urban background sites and up to 27% in traffic ones). Furthermore, though not distinguishing between different regions, the authors also reported > 20% reduction for PM_10_ at rural zones. Thus, as the contribution of traffic emissions decreased on the local and regional (with restrictions taking place in majority of European countries), PM_10_ changes at rural site might have been affected due to the lesser transport of long-distance emissions, as reported elsewhere^44^.

On the contrary, in suburban sites, in 2020 PM_10_ (Fig. 1) were higher (overall 21 µg m^–3^ vs. 20 µg m^–3^ in 2019), though these differences were not statistically significant. Specifically, the highest increases were observed in Centre (30% more in 2020) in North (20%) regions. However, Centre region consists only of 2 suburban stations and thus the respective results need to be implied carefully, possibly over longer time context. Though North region contained 77% of the suburban sites, they were background type (Table 1). In addition, on European level road transport contributes only ~ 11% of PM in EU, the main sources of PM_10_^27^ are commercial, institutional and household sector (39%) and industrial processes (20%), which could be linked with the unchanged trends of PM in the suburban zones. In terms of PM_10_ legislation, 24 h limit was exceeded in all three types of zones in 2020. However, majority of the exceedances were observed in urban zones (87%, Supplementary Table 5S) and furthermore, 80% of these exceedances occurred in January and February (i.e., before the state emergency regulations took place). In addition, it is necessary to highlight that in 2020, for the respective period of 5 months, PM_10_ daily limits were exceeded approximately 50% less (107 vs. 218 in 2019). Thus, the results indicate that PM concentrations were positively influenced in 2020, most likely also by the lower vehicle road traffic. It is necessary to highlight that Algarve was the only region that in 2020 exhibited very different evolution of PM at urban traffic sites (and higher concentrations at urban traffic sites during all 5 months of 2020) from the rest of the territory (Fig. 2a). While the previous works emphasized the impacts of long-range transport of mineral dust from North Africa with high frequency and prevalence namely in southern parts of Portugal^45,46^ it needs to be highlighted that these data (urban traffic in Algarve region) are based on 1 monitoring station (Table 1). Thus, these values will need to be confirmed when the final registry of APA is released.

Concerning the fine fraction, 2019 average daily PM_2.5_ means (Fig. 1b) were between 5 and 9 µg m^–3^ (absolute range 1–47 µg m^–3^) in rural zone of the five regions of the Continental Portugal, 9–21 µg m^–3^ (1–60 µg m^–3^) in suburban zones and 5–15 µg m^–3^ (1–53 µg m^–3^) in urban ones. In agreement with PM_10_, these results demonstrated that for all 7 regions daily PM_2.5_ concentrations were the lowest at rural zones being significantly (*p* < 0.05) different (approximately 15% in Alentejo—90% for Centre) than the respective concentration at urban zones. In 2019, for the considering period, the overall mean (7 µg m^–3^ across 68 monitoring stations) was well below the annual target (Supplementary Table 1S), though these results need to be implicated carefully, once the considered work of this study included 5 months (i.e., 42% of the calendar time). Worldwide, Portugal is among the countries with the better air quality in terms of PM_2.5_^47^; in 2017 it ranked as 7th country in European Union with the lowest PM_2.5_ across 27 members^27^. Furthermore, it is noteworthy that for fine fraction, out of the three different zones, suburban areas presented the highest PM_2.5_ (overall mean of 16 µg m^–3^) with 25–73% higher concentrations than the respective levels (of the each region) at the rural zones. Still, the exposure concentration obligation (20 µg m^–3^; calculated based on the levels of PM_2.5_ at suburban and urban background sites) is typically obliged^27,31^.

In 2020, the lowest PM_2.5_ concentrations were observed in rural zones where they ranged between 4 µg m^–3^ (Algarve) and 7 µg m^–3^ (Centre and Lisbon TV). The corresponding levels in suburban and urban areas were 30–90% higher with, respectively, overall means of 6 µg m^–3^ (Alentejo) and 12 µg m^–3^ (Lisbon TV) in suburban and 5–13 µg m^–3^ (Madeira and Centre region) in urban zones. In comparison with 2019, PM_2.5_ emissions decreased Whereas the changes of PM_2.5_ were statistically insignificant in rural zones (overall means of 6 µg m^–3^ vs. 7 µg m^–3^), the highest differences were observed in suburban (10 µg m^–3^ vs. 16 µg m^–3^ in 2019), and urban zones (8 µg m^–3^ vs. 10 µg m^–3^), being especially substantial for North (49%) and Lisbon TV region (~ 30%). In metropolitan areas, large portion of PM_2.5_ is mostly secondary origin^48^ and the atmospheric conditions might impact formation of secondary PM even if emissions of precursors are reduced^44^. In state-of-the-art study, Querol et al. assessed anomalies in pollutant concentrations across in 11 metropolitan areas of Spain^44^ and also estimated the meteorology-normalized change of several pollutants before, during and after the 2020 lockdown. The work clearly demonstrated the importance of the association between pollution changes and meteorology. In terms of PM_2.5_ the authors emphasized the potential relevance of non-vehicular regional emissions on secondary PM precursors or other emission sources such as industry, agriculture/farming. While the data for urban traffic zones in this work are limited for PM_2.5_ (Supplementary Fig. 8S), in agreement with the previous results, April was the month with the lowest concentrations. Similar to 2019, in 2020 the overall mean of PM_2.5_ was below the annual target. Fifty-one exceedances of the annual limited were registered over the 5 months in 2020 (vs. 98 exceedances in 2019, i.e., 48% less than in 2019), all of them occurring in winter months, i.e., before the restrictions were enforced, 77% in January, 22% in February.

#### Gaseous pollutants

For the gaseous pollutants the overall means of NO_2_ concentrations in 2019 (Fig. 1c) were 12 µg m^–3^ (range of Azores 3 µg m^–3^—North 16) for rural zones, 42 µg m^–3^ for suburban (33 µg m^–3^ in Centre to 64 µg m^–3^ in North) and 54 µg m^–3^ (37 µg m^–3^ in Algarve—74 µg m^–3^ in North) for urban zones. These results show the strong impact of anthropogenic emissions of level of NO_2_, being typically considered as indicator of traffic emissions^49,50^. On European level, approximately 40% of NOx emissions are contributed by road transport sector^27^. The population exposure to ambient NO_2_ concentrations is especially relevant in urban areas because its emissions are close to the ground and are distributed across densely populated areas. Furthermore, the highest concentrations of NO_2_ were observed in suburban and urban zones of North region (i.e. 40% of coverage for traffic emissions monitoring in Portugal; Table 1). Concerning the limits for heath protection, 9 exceedances of hourly limit value in 2019 were registered in 5.9% (4 stations) of all monitoring station (North and Lisbon TV region), all of them being urban sites (and 3 traffic influence).

In 2020, the means of NO_2_ concentrations at rural zones (Fig. 1c) were between 2 µg m^–3^ (Algarve) and 10 µg m^–3^ (North). In suburban zones, depending on each region the respective levels were 3–7 times higher, with means between 14 µg m^–3^ in Alentejo and 64 µg m^–3^ in North, whereas in urban zones the respective NO_2_ levels were even higher (4–18 times compared with rural zones) with range of 29–53 µg m^–3^ in Algarve and North, respectively. In agreement with the previous year, the highest levels of NO_2_ (up to 6 times) were observed for the zones (all) of North region. However, in 2020 NO_2_ levels were significantly (*p* < 0.05) lower compared with the previous year, being approximately half for the rural and 30% lower in suburban and urban zones as follows, overall mean 6 µg m^–3^ vs. 12 for rural zones, 33 µg m^–3^ vs. 43 µg m^–3^ in suburban and 43 µg m^–3^ vs. 56 µg m^–3^ in urban ones. Thus in 2020, NO_2_ pollution was significantly lower (*p* < 0.05) in all types of zones and in all regions of Portugal. NO_2_ was the pollutant with more significant changes during the two year and the restrictions associated with the COVID-19 pandemic seemed to have significant implications for relevant NO_2_ emission sources thus influencing its levels in air, both on local (direct) and regional level^43^, which might be the cause for lower concentrations of the pollutant observed at rural sites in 2020. In addition to NO_2_ emission sources and transport to other locations, chemical transformations of NO_2_ influence its ambient concentrations. After few hours in the air and in the presence of volatile organic compounds (VOCs) NO may be converted to NO_2_; with sunlight NO_2_ can convert back to NO and produce ozone^51^. Finally, meteorological conditions and surface deposition are the parameters that contribute to the temporal trends of ambient NO_2_ concentrations; March–May of 2020 was extremely warm period of year (meteorological parameters are summarized in Supplementary Table 6S), with an average temperature of 15.1 °C and several heat waves^52^, which could contribute to the respective levels. Concerning the urban traffic zones specifically (Fig. 2c), the concentrations of NO_2_ were lower than in the previous year in all regions between February and May, which might be due the annual trends. In the recent work Gama et al.^43^ assessed the air pollution trends (NO_2_ and PM_10_) during last five years (2015–2020) in Portugal, selecting approximately only half of the existent monitoring stations (34 sites) Similarly to this work, authors reported a significant drop (41%) of NO_2_ levels during the pandemic, with major changes at sites influenced by traffic. As shown in Fig. 2c significant decrease of NO_2_ levels at traffic sites was registered in March, April was then the month with the minimal means in 2020 in all the regions (range 17–33 µg m^–3^). Considering different regions, it is noteworthy that in North the NO_2_ levels were still almost twice higher (mean of 57 µg m^–3^) during the state emergency period than in the rest of country. In addition, evaluating then decrease of NO_2_ (Fig. 2d), the biggest changes between the two years were observed in March and April of 2020 when NO_2_ decreased by 15% (North) and 71% (Algarve). Considering the two largest and most populated urban areas in country (Lisbon MA and Oporto MA in Lisbon TV and North region, respectively; Supplementary Table 1S), NO_2_ cumulative decrease was 55% (40 and 15%, respectively) which from the national perspective may represent several health benefits^53^. These results clearly confirm that NO_2_ levels were significantly lowered during restrictions associated COVID-19 outbreak (especially in months of March and April). Finally, in 2020 over the period analysed in this work all monitoring stations fulfilled the limit value for the health protection and no exceedances were observed, unlike the previous year.

SO_2_ (Fig. 1d) maximum of 1 h mean concentrations ranged between 2 and 12 µg m^–3^ in rural zones, 3–13 µg m^–3^ and 3–29 µg m^–3^ in suburban and urban zones, respectively. The levels of SO_2_ were especially high in North region, where for suburban and urban zones concentrations were 3–6 times higher than in the other regions. However, across all monitoring stations, 1 h limit alert threshold (500 µg m^–3^) and 1 h limits value (350 µg m^–3^) of SO_2_ concentrations were fulfilled. In addition, in general SO_2_ levels were below the 24 h limit value; in 2019 only 3 stations (North region) registered 1 h maximum concentrations above the daily limit value, but the 24 h concentrations during those exceedances were fulfilled. In 2020 (Fig. 1d) the 1 h maximum means of SO_2_ were 5 µg m^–3^ (range of 1–10 µg m^–3^) in rural zones, and 9 µg m^–3^ (3–23 µg m^–3^) and 10 µg m^–3^ (5–18 µg m^–3^) in suburban and urban zones, respectively. For all three types of zones, the highest SO_2_ were observed in North region (up to 6 times for suburban zones and 9 times for urban ones) than in other regions. The North region was also the only one where 1 h maximum concentrations exceeded once the 24 h limit value (on urban industrial site). Finally, 1 h alert and 1 h limit was obliged in all 68 monitoring stations. Though SO_2_ is a not a pollutant associated with traffic emissions, in 2020 the overall levels were approximately 65% lower than in the period of the previous year with overall means of 5 vs. 8 µg m^–3^ which could be due to suspended industrial emissions. Nevertheless, evaluating the industrial sites specifically (suburban and urban Table 1), the means obtained between both years were not significantly different (6.3 vs. 6.9 µg m^–3^ in 2019). In addition, the monthly evolution trend did not show any change of patterns in the lock down period, however, assessment of 24 h means (oppose to 1 maximum used in this work) should be conducted when available.

Data for ozone in 2019 (Fig. 1e) that maximum 1 h mean ranged between 74 µg m^–3^ (North) and 104 µg m^–3^ (Algarve) of rural zones, 62 µg m^–3^ (North) and 90 µg m^–3^ (Alentejo) in suburban and 67 µg m^–3^ (North) and 101 µg m^–3^ (Algarve) in urban ones. These results show that registered 1 h maxima concentrations were higher (*p* < 0.05) at rural sites than those in suburban and urban ones, in agreement with other studies^54^ Production of background ozone exhibits both long-term trends and substantial annual variability^55^ also due to the variations in air-flow, air pressure or temperature^56–58^. In addition, ozone episodes are strongly influenced by meteorological conditions. The activation of photochemical reactions and efficient transport mechanisms for precursor emissions from upwind regions ideally occurs under anticyclonic conditions (i.e., the absence of cloud cover, high solar radiation, and more frequent warm temperatures^44^. Transport of ozone precursors and atmospheric chemical processes^59^ have strong impacts for ozone levels at rural zones. Firstly, NO_2_ has a longer lifespan in atmosphere (hours to days)^51^ than NO, which allows it to be transported over larger distances to rural areas, leading to NO_2_-based ozone formations^54^. Secondly, the lesser amount of NO in the atmosphere at rural sites (because of less traffic) leads to less ozone degradation. In addition, presence of VOCs at rural sites (due to emissions from vegetations) allows further reactions with NO to form NO_2_ which can form even more ozone, leading to even higher ozone concentrations. For all zones, the registered 1 h maxima of ozone were the lowest in north of country, consistently increasing towards the south, being the highest ones in southern regions of country (Algarve and Alentejo for suburban zones, Fig. 1e). In agreement, the north is the coldest part of the country, and south being the warmest one with mean air temperature as the following, 13.8 °C in North, 15.1 °C for Centre, 16.8 Lisbon TV, 16.9 °C in Alentejo and 17.0 °C in Algarve^60^. From the legislative perspective, the European hourly alert of 180 µg m^–3^ was exceeded once (North regions), whereas 1 h information threshold of 180 was reached once in Lisbon TV region (120 µg m^–3^, expressed as daily 8 h mean) though could not be clearly assessed, once the continuous measurements of ozone are not public yet. Finally, in 2020 the overall levels of ozone slightly (though not significantly) increased (83 vs. 80 µg m^–3^). The spring 2020 (Supplementary Table 6S), was considered as extremely warm one (mean temperature of 15.1 °C, total precipitation of 240 mm)^51^ which might be conducive to ozone formation. One h maxima levels ranged between 79 µg m^–3^ (North) and 92 µg m^–3^ (Alentejo) in Continental Portugal whereas levels on islands were higher (92–97 µg m^–3^). In agreement with the previous year, suburban and urban sites of Continental Portugal exhibited, respectively, significantly lower concentrations of ozone as the following, 59–80 µg m^–3^ (North and Alenetejo), and 70–80 µg m^–3^ (Centre and Lisbon TV). However, no differences were observed between the levels during the two years, with the means of 87 µg m^–3^ vs. 89 µg m^–3^ in 2020 and 2019 in rural sites, 73 µg m^–3^ vs. 79 µg m^–3^ for suburban, and 85 µg m^–3^ (in both years) at urban zones. The European hourly alert of 240 µg m^–3^ was not exceeded in 2020.

## Conclusions

This work assessed air pollution levels and trend during COVID-19 period in Portugal (January–May 2020) compared with the previous year. The issued lockdown of the country enforced by the Portuguese government resulted in some positive, yet non uniform, changes of air pollution. NO_2_ was the pollutant that showed the most consistent decrease all over the country and across all different zones of urbanizations, in accord with reduced transport. Considering that annually road transport causes between 184,00 and 242,000 premature deaths worldwide^61,62^ public health benefits from reduction of the respective emissions might be significant. Regarding particulate matter, the major decreases were observed in remote and urban zones, suburban areas were impacted to a lesser degree and most dominantly in terms of fine PM. Whereas improved air quality will persist in long-term is uncertain, nonetheless the restrictions of COVID-19 conducted on large scale and in many countries simultaneously will provide a unique opportunity to re-examine current air quality policies and possible recovery scenarios to for air pollution reduction on global level.

## Supplementary Information

Supplementary Information.

## Data Availability

The data to obtain the findings of this study were retrieved from publicly available QualAr database. The datasets analysed during the current study are available from the corresponding author on reasonable request.
