# Supplementary material for: 2020 COVID-19 lockdown and the impacts on air quality with emphasis on urban, suburban and rural zones
Source: Sci Rep. 2021 Oct 29;11:21336. doi: 10.1038/s41598-021-99491-7 (PMC8556251; doi:10.1038/s41598-021-99491-7)
Supplement: Supplementary file 1 — Supplementary Information. [file 41598_2021_99491_MOESM1_ESM.docx]

**2020 COVID-19 lockdown and the impacts on air quality with emphasis on urban, suburban and rural zones**

Klara Slezakova^a#^, Maria Carmo Pereira^a^

^a^LEPABE, Departamento de Engenharia Química, Faculdade de Engenharia, Universidade do Porto, Rua Dr. Roberto Frias, 4200-465, Porto, Portugal

#Corresponding author: e–mail: slezakok@fe.up.pt

**Table 1S**

Characterization of Portuguese territory

| Region | Area  km^2^ (%) | Population  n (%) | Population density  Inhabitants km^–2^ |
| --- | --- | --- | --- |
| North | 21 286.86  (23.1) | 3 575 338  (34.7) | 168.0 |
| Centre | 28 199.35  (30.1) | 2 217 285  (21.5) | 78.6 |
| Lisbon MA^a^ | 3 015.24  (3.3) | 2 863 272  (27.8) | 949.6 |
| Alentejo | 31 604.90  (34.3) | 704 558  (6.8) | 22.3 |
| Algarve | 4 99.7  (5.4) | 438 406  (4.3) | 87.8 |
| Madeira | 801.51  (0.9) | 254 254  (2.5) | 317.2 |
| Azores | 2 323.96  (2.5) | 242 796  (2.4) | 104.6 |
| Total | 92 226 | 10 295 909 | 111.6 |

^a^from January 1 2015, Lisbon and Tejo Valley Region with area of 11 633 km² (~12% of the total area) and population of 3 631 738 (~35%) was transformed to Lisbon Metropolitan Area^1, 2^. However, the Qualar system still uses the previous division, hence it was kept was this work.

**Table 2S**

Air pollution standards in ambient air^3^

| **Pollutant** | **Averaging time** | **Standard type** | **Value** | **Note/permitted exceedances** |
| --- | --- | --- | --- | --- |
| PM_10_ | annual | EU limit | 40 μg m^–3^ | n/a |
|  | 24 h | EU limit | 50 μg m^–3^ | not to be exceeded more than 35 times in a calendar year |
| PM_2.5_ | annual | EU limit | 25 μg m^–3^ | n/a |
|  |  | EU exposure concentration obligation | 20 μg m^–3^ | average exposure indicator (AEI)^a^ in 2015 (2013-2015 average) |
|  |  | EU national exposure reduction target | 0–20% | AEI^a^ in 2020, the percentage reduction depends on the initial AEI |
| Sulphur dioxide | 24 h | EU limit | 125 μg m^–3^ | not to be exceeded more than 3 times over a calendar year |
|  | 1 h | EU limit | 350 μg m^–3^ | not to be exceeded more than 24 times |
|  |  | EU alert threshold | 500 μg m^–3^ | to be measured over 3 consecutive hours over  100 km^2^ or an entire zone |
| Nitrogen dioxide | annual | EU limit | 40 μg m^–3^ | n/a |
|  | 1 h | EU limit | 200 μg m^–3^ | not to be exceeded more than 18 times in any calendar year |
|  |  | EU alert threshold | 400 μg m^–3^ | to be measured over 3 consecutive hours over 100 km^2^ or an entire zone |
| Ozone | maximum daily 8 h mean | EU target | 120 μg m^–3^ | not to be exceed more than 25 days per calendar year averaged over 3 years |
|  | 1 h | EU information threshold | 180 μg m^–3^ |  |
|  |  | EU alert threshold | 240 μg m^–3^ |  |
| Carbon monoxide | maximum daily 8 h mean | EU limit | 10 mg m^–3^ | n/a |
| Benzene | annual | EU limit | 5 μg m^–3^ | n/a |
| Arsenic | annual | EU target | 6 ng m^–3^ | n/a |
| Cadmium | annual | EU limit | 5 ng m^–3^ | n/a |
| Nickel | annual | EU limit | 20 ng m^–3^ | n/a |
| Lead | annual | EU limit | 0.5 μg m^–3^ | n/a |
| Polycyclic aromatic hydrocarbons^c^ | annual^a^ | EU target | 1 ng m^–3^ | n/a |

^a^AEI: based upon measurements in urban background locations established for this purpose by the Member States, assessed as a 3-year running annual mean

^b^ Measured as content in PM_10_;

^c^expressed as concentration of concentration of benzo(a)pyrene;

**Table 3S**

Share of transport (percentage) in main metropolitan areas^14^

| Mean of transport | Metropolitan Area of Oporto | Metropolitan Area of Lisbon |
| --- | --- | --- |
| Private car | 67.6 | 58.9 |
| Soft modes | 18.9 | 23.5 |
| Walking | 18.5 | 23.0 |
| Bicycle | 0.4 | 0.5 |
| Bus | 8.2 | 8.8 |
| Train | 2.8 | 6.3 |
| Motorcycle | 1.3 | 0.9 |
| Other (taxi, boat, etc.) | 1.2 | 1.6 |

**Table 4S**

Air pollution in different rural, suburban and urban zones of seven Portuguese regions: 2019 (January–May) descriptive statistics

|  | **Rural** | | | | | | | **Suburban** | | | | | | | **Urban** | | | | | | |
| --- | --- | --- | --- | --- | --- | --- | --- | --- | --- | --- | --- | --- | --- | --- | --- | --- | --- | --- | --- | --- | --- |
|  |  | | | | | | |  | | | | | | |  | | | | | | |
|  | N | C | LTV | Al | Ag | Ma | Az | N | C | LTV | Al | Ag | Ma | Az | N | C | LTV | Al | Ag | Ma | Az |
| **PM_10_** | 24 h means (µg m^–3^) | | | | | | | | | | | | | | | | | | | | |
| Mean | 12 | 18 | 20 | 17 | 17 | 11 | 7 | 18 | 18 | 21 | 19 | – | – | – | 19 | 23 | 24 |  | 20 | 19 | – |
| Min | 2 | 1 | 2 | 5 | 5 | 2 | 2 | 2 | 2 | 7 | 5 | – | – | – | 2 | 2 | 6 |  | 1 | 1 | – |
| Max | 36 | 69 | 53 | 74 | 48 | 41 | 17 | 82 | 68 | 79 | 60 | – | – | – | 92 | 70 | 99 |  | 116 | 119 | – |
| 25^th^ | 8 | 9 | 12 | 12 | 12 | 6 | 4 | 12 | 10 | 13 | 12 | – | – | – | 11 | 15 | 14 |  | 10 | 11 | – |
| 50^th^ | 10 | 15 | 17 | 16 | 16 | 9 | 6 | 16 | 15 | 20 | 17 | – | – | – | 17 | 20 | 19 |  | 16 | 15 | – |
| 75^th^ | 14 | 23 | 23 | 20 | 21 | 14 | 8 | 22 | 22 | 26 | 23 | – | – | – | 23 | 27 | 28 |  | 26 | 21 | – |
| Total exceedance  N (Min-Max)^a^ | – | 25  (6-18) | 32  (4-20) | 1 | – | – | – | 26  (0-11) | 11 | 3  – | 11  6 | – | – | – | 12  (0-7) | 14  (4-6) | 61  (1-8) | – | 12  (5-7) | 15  (7-8) | – |
| **PM_2.5_** | 24 h means (µg m^–3^) | | | | | | | | | | | | | | | | | | | | |
| Mean | – | 7 | 9 | 5 | 6 | 3 | 3 | – | 9 | 21 | 19 | – | – | – | 11 | 13 | 9 | 6 | 8 | 5 | – |
| Min | – | 2 | 1 | 1 | 3 | 1 | 1 | – | 1 | 7 | 5 | – | – | – | 1 | 2 | 1 | 1 | 1 | 1 | – |
| Max | – | 47 | 38 | 23 | 29 | 11 | 7 | – | 54 | 58 | 60 | – | – | – | 33 | 53 | 51 | 17 | 27 | 30 | – |
| 25^th^ | – | 4 | 5 | 3 | 4 | 2 | 2 | – | 3 | 13 | 12 | – | – | – | 4 | 6 | 5 | 2 | 3 | 2 | – |
| 50^th^ | – | 6 | 7 | 5 | 7 | 3 | 2 | – | 5 | 19,5 | 17 | – | – | – | 8 | 10 | 8 | 5 | 5 | 4 | – |
| 75^th^ | – | 11 | 11 | 8 | 9 | 4 | 3 | – | 10 | 26 | 23 | – | – | – | 17 | 17 | 12 | 7 | 7 | 6 | – |
| **SO_2_** | 1 h maximum (µg m^–3^) | | | | | | | | | | | | | | | | | | | |  |
| Mean | 8 | 12 | 5 | 8 | 16 | 2 | 2 | 13 | 3 | 3 | 4 | – | – | – | 19 | 5 | 3 | – | 29 | 4 | – |
| Min | 3 | 1 | 1 | 1 | 2 | 1 | 1 | 2 | 2 | 1 | 1 | – | – | – | 4 | 1 | 1 | – | 25 | 1 | – |
| Max | 23 | 44 | 26 | 12 | 32 | 5 | 4 | 55 | 12 | 20 | 13 | – | – | – | 212 | 65 | 18 | – | 34 | 28 | – |
| 25^th^ | 7 | 4 | 2 | 3 | 10 | 2 | 2 | 6 | 2 | 2 | 3 | – | – | – | 6 | 2 | 2 | – | 27 | 2 | – |
| 50^th^ | 8 | 10 | 3 | 8 | 18 | 2 | 2 | 7 | 2 | 2 | 3 | – | – | – | 7 | 3 | 3 | – | 28 | 3 | – |
| 75^th^ | 9 | 18 | 5 | 9 | 23 | 3 | 3 | 15 | 3 | 3 | 4 | – | – | – | 13 | 7 | 4 | – | 30 | 5 | – |
| Exceedance 24h  n (Min-Max)^a^ | – | – | – | – | – | – | – | – | – | – | – | – | – | – | 3  (0-3) | – | – | – | – | – | – |
| Exceedance 1h  n (Min-Max)^a^ | – | – | – | – | – | – | – | – | – | – | – | – | – | – | – | – | – | – | – | – | – |
| **NO_2_** | 1 h maximum (µg m^–3^) | | | | | | | | | | | | | | | | | | | |  |
| Mean | 16 | 15 | 23 | 12 | 9 | 4 | 3 | 64 | 32 | 45 | 33 | – | – | – | 74 | 58 | 56 | – | 37 | 45 | – |
| Min | 1 | 1 | 4 | 2 | 1 | 1 | 2 | 8 | 5 | 8 | 2 | – | – | – | 5 | 6 | 2 | – | 1 | 2 | – |
| Max | 39 | 58 | 52 | 20 | 14 | 56 | 13 | 175 | 91 | 102 | 105 | – | – | – | 228 | 166 | 231 | – | 129 | 147 | – |
| 25^th^ | 10 | 6 | 19 | 4 | 5 | 2 | 2 | 34 | 20 | 26 | 6 | – | – | – | 35 | 20 | 19 | – | 4 | 7 | – |
| 50^th^ | 25 | 11 | 26 | 8 | 7 | 3 | 2 | 58 | 28 | 41 | 20 | – | – | – | 62 | 53 | 39 | – | 32 | 34 | – |
| 75^th^ | 30 | 29 | 31 | 16 | 8 | 5 | 3 | 90 | 39 | 63 | 62 | – | – | – | 109 | 90 | 72 | – | 60 | 79 | – |
| Exceedance 24h  n (Min-Max)^a^ | – | – | – | – | – | – | – | – | – | – | – | – | – | – | 4  (0-2) | – | 5  (0-2) | – |  |  | – |
| **O_3_** | 1 h maximum (µg m^–3^) | | | | | | | | | | | | | | | | | | | | |
| Mean | 74 | 86 | 89 | 95 | 104 | 88 | 84 | 62 | 78 | 86 | 90 | – | – | – | 67 | 70 | 85 | – | 102 | 101 |  |
| Min | 19 | 29 | 14 | 42 | 61 | 47 | 43 | 11 | 11 | 31 | 55 | – | – | – | 11 | 21 | 11 | – | 57 | 66 |  |
| Max | 183 | 144 | 166 | 136 | 147 | 137 | 118 | 115 | 134 | 139 | 140 | – | – | – | 179 | 116 | 145 | – | 154 | 154 |  |
| 25^th^ | 56 | 78 | 72 | 87,25 | 89,25 | 78 | 74 | 48 | 63 | 75 | 75,25 | – | – | – | 52 | 59 | 72 | – | 91 | 88 |  |
| 50^th^ | 75 | 88 | 88 | 95 | 103 | 88 | 88 | 61 | 80,5 | 87 | 92 | – | – | – | 64 | 69 | 85 | – | 105 | 97 |  |
| 75^th^ | 89 | 97 | 99 | 104 | 115 | 99 | 97 | 74 | 92 | 98 | 103 | – | – | – | 79 | 80 | 98 |  | 116 | 112 |  |
| Exceedance  1h alert | – | – | – | – | – | – | – | – | – | – | – | – | – | – | – | – | – | – | – | – | – |

Abbreviation: N=North, C= Centre, LTV=Lisbon and Tejo Valley, Ag= Algarve, Aj= Alentejo, M= Madeira, Az=Azores

Note: ^a^Minimal and maximal number of limit exceedances registered per individual monitoring station

**Table 5S**

Air pollution in different rural, suburban and urban zones of seven Portuguese regions: 2020 (January–May) descriptive statistics

|  | **Rural** | | | | | | | **Suburban** | | | | | | | **Urban** | | | | | | |
| --- | --- | --- | --- | --- | --- | --- | --- | --- | --- | --- | --- | --- | --- | --- | --- | --- | --- | --- | --- | --- | --- |
|  |  | | | | | | |  | | | | | | |  | | | | | | |
|  | N | C | LTV | Al | Ag | Ma | Az | N | C | LTV | Al | Ag | Ma | Az | N | C | LTV | Al | Ag | Ma | Az |
| **PM_10_** | 24 h means (µg m^–3^) | | | | | | | | | | | | | | | | | | | | |
| Mean | 8 | 13 | 15 | 17 | 10 | 14 | 10 | 21 | 24 | 22 | 17 | – | – | – | 19 | 18 | 19 | – | 25 | 18 | – |
| Min | 2 | 1 | 1 | 1 | 1 | 1 | 2 | 1 | 2 | 4 | 2 | – | – | – | 2 | 2 | 3 | – | 2 | 2 | – |
| Max | 35 | 100 | 51 | 83 | 94 | 111 | 34 | 68 | 90 | 75 | 118 | – | – | – | 84 | 67 | 66 | – | 166 | 121 | – |
| 25^th^ | 5 | 5 | 9 | 7 | 4 | 7 | 6 | 12 | 14 | 14 | 11 | – | – | – | 11 | 11 | 12 | – | 13 | 10 | – |
| 50^th^ | 8 | 10 | 14 | 10 | 7 | 11 | 8 | 21 | 22 | 19 | 15 | – | – | – | 17 | 16 | 17 | – | 19 | 14 | – |
| 75^th^ | 10 | 17 | 19 | 19 | 11 | 16 | 13 | 27 | 29 | 28 | 21 | – | – | – | 24 | 24 | 24 | – | 28 | 20 | – |
| Total exceedance  N (Min-Max)^a^ | 0 | 3  (1-2) | 1  – | 3  (1-1) | 3  – | 3  – | 0 | 9  (1-4) | 19  (9-10) | 8  – | 1  – | – | – | – | 9  (1-4) | 11  (2-7) | 27  (1-4) | – | 22  (3-16) | 15  (6-9) | – |
| **PM_2.5_** | 24 h means (µg m^–3^) | | | | | | | | | | | | | | | | | | | | |
| Mean | – | 7 | 7 | 4 | 6 | 4 | 6 | – | 12 | 12 | 6 | – | – | – | 6 | 13 | 11 | 6 | 8 | 5 | – |
| Min | – | 1 | 1 | 1 | 1 | 1 | 1 | – | 2 | 3 | 1 | – | – | – | 1 | 2 | 1 | 1 | 3 | 1 | – |
| Max | – | 36 | 23 | 19 | 26 | 32 | 29 | – | 56 | 63 | 25 | – | – | – | 19 | 53 | 38 | 17 | 23 | 30 | – |
| 25^th^ | – | 3 | 3 | 2 | 4 | 2 | 4 | – | 5 | 6 | 3 | – | – | – | 2 | 6 | 6 | 2 | 5 | 3 | – |
| 50^th^ | – | 5 | 6 | 4 | 5 | 3 | 5 | – | 10 | 10 | 5 | – | – | – | 4 | 10 | 9 | 5 | 8 | 4 | – |
| 75^th^ | – | 10 | 9 | 6 | 7 | 5 | 7 | – | 15 | 13 | 8 | – | – | – | 9 | 17 | 14 | 7 | 10 | 7 | – |
| **SO_2_** | 1 h maximum (µg m^–3^) | | | | | | | | | | | | | | | | | | | |  |
| Mean | 10 | 3 | 6 | 4 | 5 | 1 | 3 | 23 | 6 | 6 | 3 | – | – | – | 18 | – | 3 | – | 15 | 5 | – |
| Min | 5 | 1 | 1 | 2 | 2 | 1 | 1 | 2 | 1 | 1 | 2 | – | – | – | 2 | – | 1 | – | 14 | 1 | – |
| Max | 18 | 6 | 92 | 5 | 8 | 3 | 9 | 150 | 17 | 47 | 4 | – | – | – | 55 | – | 25 | – | 16 | 32 | – |
| 25^th^ | 6 | 2 | 3 | 3 | 3 | 1 | 2 | 10 | 3 | 3 | 2 | – | – | – | 10 | – | 2 | – | 15 | 2 | – |
| 50^th^ | 10 | 3 | 5 | 3 | 5 | 1 | 3 | 12 | 5 | 5 | 3 | – | – | – | 11 | – | 3 | – | 15 | 3 | – |
| 75^th^ | 14 | 4 | 7 | 4 | 6 | 2 | 4 | 36 | 8 | 7 | 3 | – | – | – | 30 | – | 4 | – | 15 | 5 | – |
| Exceedance 24h  n (Min-Max)^a^ | – | – | – | – | – | – | – | 1 | – | – | – | – | – | – | 0) | – | – | – | – | – | – |
| Exceedance 1h  n (Min-Max)^a^ | – | – | – | – | – | – | – | – | – | – | – | – | – | – | – | – | – | – | – | – | – |
| **NO_2_** | 1 h maximum (µg m^–3^) | | | | | | | | | | | | | | | | | | | |  |
| Mean | 10 | 6 | 9 | 5 | 2 | 4 | 3 | 64 | 17 | 37 | 14 | – | – | – | 53 | 38 | 43 | – | 29 | 52 | – |
| Min | 1 | 1 | 2 | 2 | 1 | 1 | 1 | 8 | 5 | 8 | 1 | – | – | – | 6 | 5 | 4 | – | 1 | 3 | – |
| Max | 22 | 39 | 51 | 20 | 15 | 24 | 9 | 175 | 73 | 100 | 66 | – | – | – | 187 | 121 | 200 | – | 105 | 144 | – |
| 25^th^ | 5 | 3 | 6 | 3 | 1 | 2 | 2 | 34 | 10 | 22 | 6 | – | – | – | 27 | 21 | 20 | – | 11 | 29 | – |
| 50^th^ | 10 | 5 | 8 | 4 | 1 | 3 | 2 | 58 | 14 | 30 | 12 | – | – | – | 46 | 33 | 36 | – | 22 | 54 | – |
| 75^th^ | 13 | 7 | 13 | 6 | 2 | 5 | 3 | 90 | 21 | 50 | 19 | – | – | – | 70 | 52 | 59 | – | 45 | 81 | – |
| Exceedance 24h  n (Min-Max)^a^ | – | – | – | – | – | – | – | – | – | – | – | – | – | – | – | – | – | – | – | – |  |
| **O_3_** | 1 h maximum (µg m^–3^) | | | | | | | | | | | | | | | | | | | | |
| Mean | 79 | 84 | 84 | 92 | 83 | 92 | 97 | 59 | 76 | 75 | 80 | – | – | – | 79 | 70 | 80 |  | 91 | 106 | – |
| Min | 25 | 35 | 40 | 51 | 57 | 49 | 36 | 1 | 36 | 15 | 40 | – | – | – | 8 | 28 | 4 |  | 48 | 36 | – |
| Max | 149 | 241 | 180 | 133 | 110 | 127 | 121 | 108 | 155 | 129 | 134 | – | – | – | 173 | 117 | 174 |  | 131 | 133 | – |
| 25^th^ | 61 | 71 | 73 | 76 | 77 | 75 | 92 | 48 | 62 | 64 | 65 | – | – | – | 65 | 61 | 69 |  | 82 | 102 | – |
| 50^th^ | 82 | 81 | 84 | 89 | 82 | 95 | 99 | 60 | 74 | 78 | 76 | – | – | – | 80 | 72 | 82 |  | 91 | 109 | – |
| 75^th^ | 94 | 92 | 93 | 99 | 90 | 108 | 104 | 75 | 89 | 87 | 92 | – | – | – | 93 | 81 | 92 |  | 100 | 1156 | – |
| Exceedance  1h alert | – | – | – | – | – | – | – | – | – | – | – | – | – | – | – | – | – | – | – | – | – |

Abbreviation: N=North, C= Centre, LTV=Lisbon and Tejo Valley, Ag= Algarve, Aj= Alentejo, M= Madeira, Az=Azores

Note: ^a^Minimal and maximal number of limit exceedances registered per individual monitoring station

**Table 6S**

Meteorological conditions (air temperature – T; total precipitation) in Portuguese territory during the time of the study^4-13^

| Region | T_Min–Max_ (°C) | | | | |  | Total precipitation (mm) | | | | |
| --- | --- | --- | --- | --- | --- | --- | --- | --- | --- | --- | --- |
|  | January | February | March | April | May |  | January | February | March | April | May |
| North | 1.4-14.9 | 3.5-18.2 | 4.4-18.5 | 7.4-19.4 | 10.9-26.3 |  | 65.1-254.1 | 5.7-108.5 | 85.8-134.9 | 120.9-196.9 | 32.7-105.8 |
| Centre | 2.8-15.4 | 5.6-17.6 | 3.9-17.7 | 5.9-18.7 | 11.7-25.8 |  | 43.5-121.7 | 2.7-37.6 | 67.2-124.2 | 96.1-240.4 | 49.7-78.2 |
| Lisbon MA^a^ | 8.9-14.8 | 10.1-18.6 | 10.5-18.6 | 11.7-19.2 | 15.4-25.4 |  | 52.8 | 8.4 | 15.6 | 139.4 | 53.9 |
| Alentejo | 5.3-15.8 | 6.0-20.1 | 3.9-20.0 | 8.8-19.8 | 12.1-27.3 |  | 43.5-83.8 | 3.9-20.1 | 20.1-81.4 | 111.4-132.3 | 43.3-92.8 |
| Algarve | 6.5-16.5 | 7.9-19.5 | 7.2-19.4 | 9.5-20.1 | 12.8-28.0 |  | 29.6-39.8 | 0.7-1.2 | 43.1-75.1 | 83.0-95.8 | 37.4-46.0 |
| Continent  mean (range) | 9.58  5.3-19.9 | 12.43  7.0-17.9 | 12.33  6.9-17.7 | 13.91  9.3-18.6 | 19.0  12.6-25.4 |  | 76.0  – | 16.6  – | 71.9  – | 117.1  – | 51.2  – |
| Azores | 5.7-21.5 | 7.6-24.0 | 6.7-23.0 | 8.6-22.2 | 9.8-25.8 |  | 76.6-397.2 | 17.9-227.6 | 22.9-107.4 | 71.8-140.3 | 38.0-151.0 |
| Note: Temperature range represents the average minima and average maxima across different stations of each region;  Precipitations is expressed as a range of averages values across different stations of each region. | | | | | | | | | | | |

**Figure 1S**

Portuguese air pollution monitoring network: abundance of zone- and emission influence-specific monitoring sites. Note: The territory is composed of five regions for continental Portugal (North, Centre, Lisbon and Tejo Valley, Alentejo, and Algarve) and two in Portuguese islands (Madeira, and Azores).

Note: the specific definition of the monitoring site is: • traffic – situated in a close proximity to a single major road; • industrial stations — located in close proximity to an industrial area or an industrial source; • background stations — pollution levels are representative of the average exposure of the general population or vegetation. Depending on the distribution/density of buildings, the area surrounding the station is classified as follows: • urban — continuously built-up urban area; • suburban — largely built-up urban area; • rural — all other areas.

**Figure 2S**

Quarterly evolution of average daily traffic (motorways) in Portugal in 2018-2020^15, 16^. Note: 2^nd^ quarter of 2020 represented a drop of 32% when compared with 1^st^ quarter of that year. Traffic in 2^nd^ quarter of 2020 was 46 and 49 % lower when compared with years 2018 and 2019, respectively.

**Figure 3S**

The trends of community movement (1.3.-31.5.2020) in Portugal across various sectors.


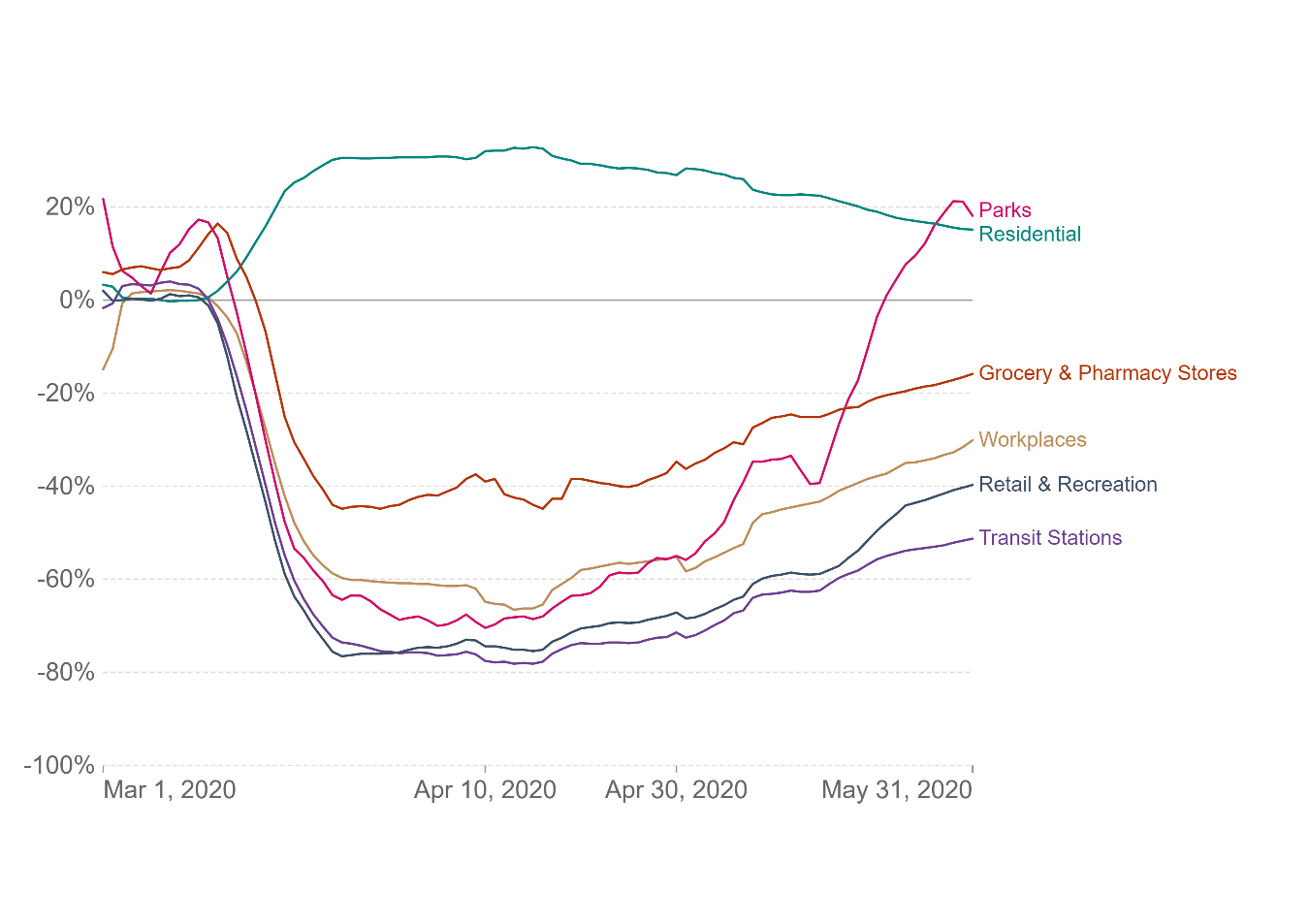
 Note: Information was adapted from Google Mobility reports^17^. The ‘Residential’ category shows a change in “duration of time” spent at home; other categories indicate a change in total visitors.

**Figure 4S**

Composition of traffic (on motorways) in Portugal^16, 17^: (a) in 2019, and (b) 2020.

|  |  |
| --- | --- |
| (a) | (b) |

**Figure 5S**

Selected comparisons of daily variations of pollutants during January – May 2020 at different sites: a) PM_10_, b) PM_2.5_; and c) NO_2_.

| . |
| --- |
| (a) |
|  |
| (b) |
|  |
| (c) |

Note: representations of the daily variations are averaged across all the stations of that type in the respective region.

**Figure 6S**

Temporal concentrations of pollutants: examples of daily variations in 2019 *vs*. 2020: a) PM_10_, b) PM_2.5_; and c) NO_2_. Note: Profiles represent examples of daily concentration trends at the same station (urban-traffic) in 2019 and 2020. The vertical lines indicate the beginning and end of implemented lock-down (*i.e.,* state of emergency) in 2020.

| (a) |
| --- |
| 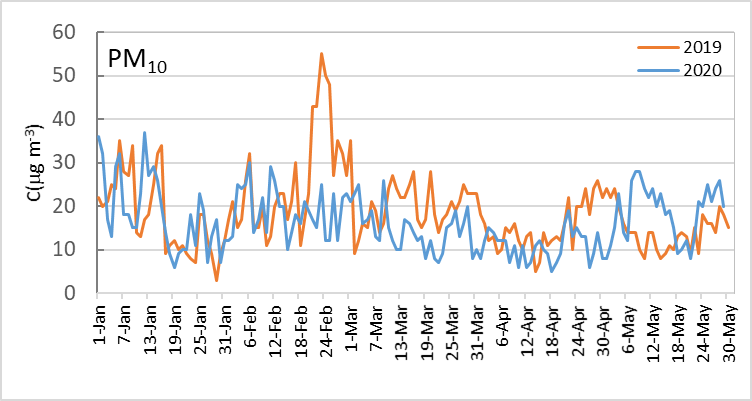 |
| (b) |
| 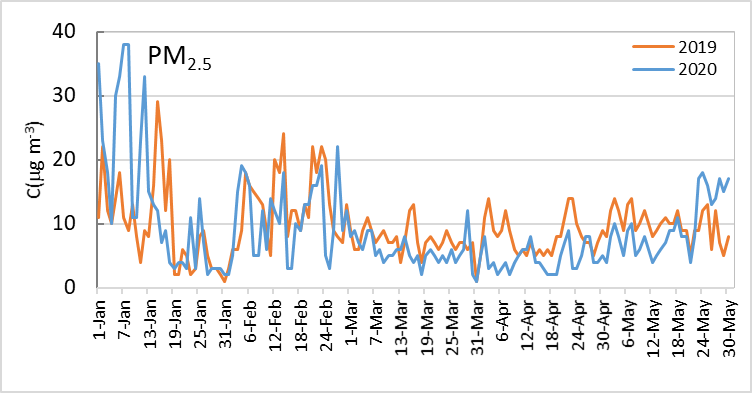 |
| (c) |
| 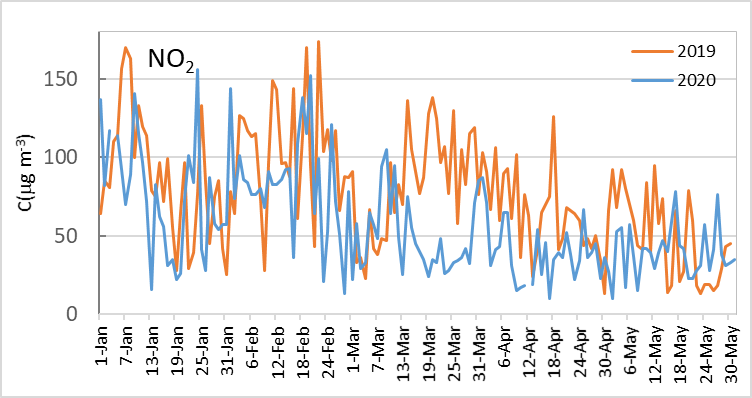 |

**Figure 7S**

Temporal profiles of pollutants in 2019 *vs* 2020: a) PM_10_, b) PM_2.5_; and c) NO_2_. Concentrations are averaged across all urban traffic stations in the respective region in 2019 and 2020. The vertical lines indicate the beginning and end of implemented lock-down (*i.e.,* state of emergency) in 2020.

| (a) |
| --- |
| 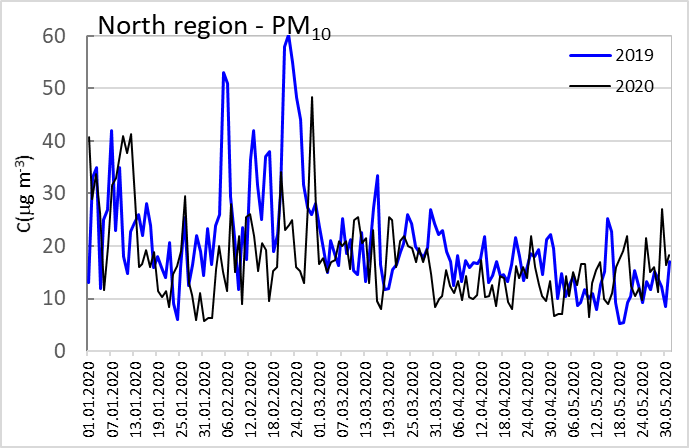 |
| (b) |
|  |
| (c) |
| 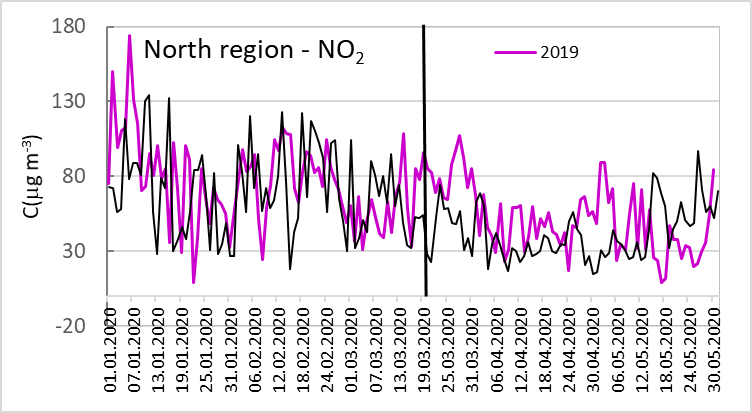 |

**Figures 8S**

Assessment of traffic related PM_2.5_ in Portugal: (a) monthly (January – May) evolution in 2019 (continuous line) and 2020 (dashed line); (b) representation of concentration changes in 2020 *vs.* 2019. Note: 22 urban/traffic monitoring stations were considered but no PM_2.5_ data exited for in 2020 for Centre and Algarve regions in 2020, and North (both years).

(a)

(b)

**References**

[1] Law11-A/2013. Reorganização administrativa do território das freguesia. Diário da República 19/2013, 1º Suplemento, 552-(2) – 552-(147) (2013).

[2] Law 56/2012. Reorganização administrativa de Lisboa. Diário da República n.º 216/2012, Série I , 6454 – 6460 (2012).

[3] European Parliament. Directive 2008/50/EC of the European Parliament and of the Council on ambient air quality and cleaner air for Europe. *Official Journal of European Union* **L152**, 1–44 (2007).

[4] Instituto Português do Mar e da Atmosfera. Boletim Climatológico, Janeiro 2020, Portugal Continental (in Portuguese). Instituto Português do Mar e da Atmosfera, I.P., Divisão de Clima e Alterações Climáticas, Lisbon, Portugal. Accessed in August 2021, available at <https://www.ipma.pt/resources.www/docs/im.publicacoes/edicoes.online/20200214/BEGzqweyksCjzjUAIArQ/cli_20200101_20200131_pcl_mm_co_pt.pdf> (2020).

[5] Instituto Português do Mar e da Atmosfera. Boletim Climatológico, Fevereiro 2020, Portugal Continental (in Portuguese). Instituto Português do Mar e da Atmosfera, I.P., Divisão de Clima e Alterações Climáticas, Lisbon, Portugal. Accessed in August 2021, available at <https://www.ipma.pt/resources.www/docs/im.publicacoes/edicoes.online/20200317/YDtMPXJqwCRMxZXdHKhI/cli_20200201_20200229_pcl_mm_co_pt.pdf> (2020).

[6] Instituto Português do Mar e da Atmosfera. Boletim Climatológico, Março 2020, Portugal Continental (in Portuguese). Instituto Português do Mar e da Atmosfera, I.P., Divisão de Clima e Alterações Climáticas, Lisbon, Portugal. Accessed in August 2021, available at <https://www.ipma.pt/resources.www/docs/im.publicacoes/edicoes.online/20200414/TpvHRgQAbBhQFDygCNSr/cli_20200301_20200331_pcl_mm_co_pt.pdf> (2020).

[7] Instituto Português do Mar e da Atmosfera. Boletim Climatológico, Abril 2020, Portugal Continental (in Portuguese). Instituto Português do Mar e da Atmosfera, I.P., Divisão de Clima e Alterações Climáticas, Lisbon, Portugal. Accessed in August 2021, svailable at <https://www.ipma.pt/resources.www/docs/im.publicacoes/edicoes.online/20200507/oCTuTXeIUTsZKCvKOJOP/cli_20200401_20200430_pcl_mm_co_pt.pdf> (2020).

[8] Instituto Português do Mar e da Atmosfera. Boletim Climatológico, Maio 2020, Portugal Continental (in Portuguese). Instituto Português do Mar e da Atmosfera, I.P., Divisão de Clima e Alterações Climáticas, Lisbon, Portugal. Accessed in August 2021, available at <https://www.ipma.pt/resources.www/docs/im.publicacoes/edicoes.online/20200617/ikazmLBHcRAEJjYONHBw/cli_20200501_20200531_pcl_mm_co_pt.pdf> (2020).

[9] Instituto Português do Mar e da Atmosfera. Boletim Climatológico Mensal dos Açores - Janeiro de 2020, (in Portuguese). Instituto Português do Mar e da Atmosfera, I.P., Divisão de Clima e Alterações Climáticas, Lisbon, Portugal. Accessed in August 2021, available at <https://www.ipma.pt/resources.www/docs/im.publicacoes/edicoes.online/20200306/yYeRpdgXJxbhimRLCJYv/cli_20200101_20200131_pcl_mm_az_pt.pdf> (2020).

[10] Instituto Português do Mar e da Atmosfera. Boletim Climatológico Mensal dos Açores - Fevereiro de 2020, (in Portuguese). Instituto Português do Mar e da Atmosfera, I.P., Divisão de Clima e Alterações Climáticas, Lisbon, Portugal. Accessed in August 2021, available at <https://www.ipma.pt/resources.www/docs/im.publicacoes/edicoes.online/20200318/cVwxsEGWIHWPklRBPLyA/cli_20200201_20200229_pcl_mm_az_pt.pdf> (2020).

[11] Instituto Português do Mar e da Atmosfera. Boletim Climatológico Mensal dos Açores - Março de 2020, (in Portuguese). Instituto Português do Mar e da Atmosfera, I.P., Divisão de Clima e Alterações Climáticas, Lisbon, Portugal. Accessed in August 2021, available at <https://www.ipma.pt/resources.www/docs/im.publicacoes/edicoes.online/20200414/knfBFKtTjOXctXgojklY/cli_20200301_20200331_pcl_mm_az_pt.pdf> (2020).

[12] Instituto Português do Mar e da Atmosfera. Boletim Climatológico Mensal dos Açores - Abril de 2020, (in Portuguese). Instituto Português do Mar e da Atmosfera, I.P., Divisão de Clima e Alterações Climáticas, Lisbon, Portugal. Accessed in August 2021, available at <https://www.ipma.pt/resources.www/docs/im.publicacoes/edicoes.online/20200506/JTlgSRrNStRyDyoLQrbA/cli_20200401_20200430_pcl_mm_az_pt.pdf> (2020).

[13] Instituto Português do Mar e da Atmosfera. Boletim Climatológico Mensal dos Açores - Maio de 2020, (in Portuguese). Instituto Português do Mar e da Atmosfera, I.P., Divisão de Clima e Alterações Climáticas, Lisbon, Portugal. Accessed in August 2021, available at <https://www.ipma.pt/resources.www/docs/im.publicacoes/edicoes.online/20200620/aVwTiDGWNSMoMXHeffpl/cli_20200501_20200531_pcl_mm_az_pt.pdf> (2020).

[14] Statistics Portugal. Mobilidade e funcionalidade do território nas Áreas Metropolitanas do Porto e de Lisboa: 2017 (in Portugesse). Instituto Nacional de Estatística, Lisbon, Portugal (2018).

[15] Brisa Concessão Rodoviária. FY 2019 Traffic Update. IRISA Concessão Rodoviária, S.A., published on 29^th^ January 2020, retrieved from https://www.brisaconcessao.pt/Portals/0/comunicados/EN/BCR%202019%20Traffic%20Update_v2.pdf, <https://web3.cmvm.pt/sdi/emitentes/docs/FR75326.pdf> (2020).

[16] Brisa Concessão Rodoviária. Resultados 2020 (in Portuguese). BRISA Concessão Rodoviária, S.A, published on 10^th^ February 2020, retrieved from https://www.brisaconcessao.pt/Portals/0/relat%F3rios/BCR%202020%20Apres%20Resultados%20PT.pdf, (2021).

[17] Ritchie, H. et al. Coronavirus Pandemic (COVID-19). Published online at OurWorldInData.org. Retrieved from: https://ourworldindata.org/coronavirus (2020).
